# Prevalence and determinants of readmission at a tertiary psychiatric hospital in Zanzibar

**DOI:** 10.4102/sajpsychiatry.v32i0.2694

**Published:** 2026-10-07

**Authors:** Abdallah K. Abdallah, Theresia A. Ottaru, Candida Moshiro

**Affiliations:** 1 Department of Epidemiology and Biostatistics, School of Public Health and Social Sciences, Muhimbili University of Health and Allied Sciences (MUHAS), Dar es Salaam, United Republic of Tanzania; 2 Department of Nursing and Midwifery, Zanzibar School of Health (ZSH), Zanzibar, United Republic of Tanzania

**Keywords:** psychiatric readmission, prevalence, determinants, mental disorders, psychiatric patients, Zanzibar

## Abstract

**Background:**

Psychiatric readmission is a global concern, leading to poorer clinical outcomes and higher healthcare costs. In Tanzania, where mental health services operate within a resource-constrained health system, repeated psychiatric admissions may place additional pressure on already limited services. However, local evidence on psychiatric readmission remains limited.

**Aim:**

To determine the prevalence of psychiatric readmission and its determinants among psychiatric patients admitted to a tertiary psychiatric hospital in Unguja, Zanzibar.

**Setting:**

A tertiary psychiatric referral hospital in Unguja, Zanzibar, serving patients across Unguja and Pemba islands.

**Methods:**

A cross-sectional study was conducted among psychiatric patients 18 years and older admitted between June 2024 and August 2024. Data were collected through interviewer-administered questionnaires. Modified Poisson regression with robust error variance was used to identify factors associated with psychiatric readmission.

**Results:**

A total of 311 patients (mean age 36, standard deviation [s.d.] = 11 years; females 52.4%; schizophrenia 32.5%) were enrolled. The prevalence of psychiatric readmission within 3 months was 58.5% (n = 182). Readmission was associated with age group 30–39 years (adjusted prevalence ratio [APR] = 1.47; 95% confidence interval [CI]: 1.16–1.84, *p*-value [*p*] = 0.001), schizophrenia (APR = 1.23; CI: 1.00–1.50, *p* = 0.041), bipolar disorders (APR = 1.69; CI: 1.26–2.26, *p* < 0.001), higher education (APR = 1.61; CI: 1.16–2.22, *p* = 0.010), history of physical abuse (APR = 1.49; CI: 1.16–2.22, *p* < 0.001), and being prescribed both oral and injectable medications (APR = 1.46; CI: 1.37–2.20, *p* < 0.001).

**Conclusion:**

There is a high psychiatric readmission rate in Zanzibar. Interventions should focus on high-risk groups, particularly middle-aged individuals and those requiring combination treatment to break the cycle of readmission and promote long-term mental health recovery.

**Contribution:**

This study provides evidence on psychiatric readmission in Zanzibar and identifies high-risk groups requiring targeted post-discharge intervention in low-resource psychiatric settings.

## Introduction

Globally, an estimated 970 million people are living with mental disorders, resulting in significant economic burdens on families, the healthcare system and individuals themselves.^1^ The disability-adjusted life years (DALYs) of mental disorders are estimated at 2375.2 in France, 1709 in Kenya and 1721 in Tanzania per 100 000 population,^2,3,4^ indicating a consistently high burden across both high-income and low- and middle-income countries.

Despite the growing burden of mental health disorders, mental health services are severely underfunded worldwide. It is estimated that only 2.5% of total government health expenditure is allocated to mental health worldwide, and as little as 0.1% in Africa.^4^ In most low- and middle-income countries, including Tanzania, less than 5% of health budgets are allocated to mental health,^2,3,4^ limiting timely and appropriate psychiatric care and not meeting recommended guidelines.^5^ Limited funding and inadequate mental health services increase the risks of persistent symptoms, complications and post-discharge challenges like low self-esteem and coping difficulties that drive frequent readmissions.^6,7^

Psychiatric readmission refers to the re-hospitalisation of a patient within a specified period following discharge from a psychiatric inpatient admission.^7,8,9^ The prevalence of psychiatric readmission is 23.6% in China,^10^ 19.21% in South Africa^11^ and 23% in Uganda,^12^ suggesting that readmission remains a persistent challenge across both high-income and low- and middle-income countries.

Psychiatric readmission is a multifactorial outcome. Socio-demographic determinants such as age, gender, marital status, social support and stressful life events have been linked to relapse.^13,14,15,16,17,18,19,20^ Studies have shown higher readmission among patients aged 30–39 years, single males, those with inadequate family or social support, and individuals exposed to physical assault.^14,16,17,18,19,20^ Behavioural factors including non-adherence to medication, dysfunctional relationships and substance use further increase the likelihood of readmission, particularly among youth and patients with schizophrenia.^13,14,15,16^

Psychiatric readmissions have significant consequences, such as reduced quality of life for patients, and emotional and financial strain on families and health care system.^8,21,22,23^ In Tanzania, mental health services are constrained by limited resources and funding, recurrent psychiatric admissions may exacerbate existing service demands and further strain the capacity of healthcare facilities.^4,24^ However, data on the prevalence and factors associated with psychiatric readmission remain limited. Understanding the prevalence of psychiatric readmission and its associated factors is important for informing measures to improve psychiatric care and reduce healthcare costs.

## Research methods and design

### Study design

A hospital-based cross-sectional study was conducted between June 2024 and August 2024. This design was considered appropriate because it enabled the collection of data on psychiatric readmission and its determinants from the study population at a single point in time.

### Setting

The study was conducted at a tertiary-level psychiatric facility in Zanzibar, which serves as the referral centre for all mental health cases across the islands (Unguja and Pemba). It provides both inpatient and outpatient psychiatric services, including acute and sub-acute care, detoxification, paediatric psychiatric care, rehabilitation and follow-up services. The hospital accommodates over 900 patients annually, with 65–90 admissions per month.

According to hospital management, no formal written hospital guidelines for inpatient psychiatric care or post-discharge care were available during the study period. However, caregivers were expected to be present at ward rounds at the time of discharge to accompany the patient home and receive follow-up plans and aftercare instructions. In addition to the above services, caregivers are provided with health education sessions aimed at equipping them with knowledge and skills to better support the recovery of their loved ones at home. Counselling sessions are also offered to clients after discharge as part of ongoing therapeutic support to address emotional and psychological challenges. Furthermore, occupational therapy (OT) sessions are conducted 2 days a week to help patients develop or regain functional skills for daily living, improve coping strategies and promote overall mental well-being.

### Study population and sampling

Eligible participants were psychiatric patients aged 18 years and older admitted to the psychiatric ward during the study period (June 2024 and August 2024) and had a minimum length of stay of 2 days. The 2-day duration was chosen to distinguish true inpatient psychiatric readmissions from brief observation visits that may not represent clinically meaningful readmissions.^9,25,26^ Admitted patients were not eligible if admitted for non-psychiatric reasons such as palliative care or for legal assessment following alleged criminal offences without a confirmed psychiatric diagnosis, unable or unwilling to provide informed consent.

The sample size was calculated assuming a 50% prevalence of psychiatric readmission because of the absence of an appropriate proportion in a similar context, with a 95% confidence level and a 6% margin of error. After considering a 10% non-response rate, the desired sample size was 300 participants. Eligible patients were enrolled as they became available until the desired sample size was achieved. For patients with multiple readmissions during the study period, only the first admission was considered.

### Data collection

Data were collected through interviewer-administered structured questionnaires. Eligible patients were identified from the admission register and screened according to the inclusion and exclusion criteria. Following clinical stabilisation and prior to discharge, eligible participants and their primary caregivers were approached, provided with information about the study, and written informed consent was obtained from the caregivers. Trained research assistants conducted face-to-face interviews with patients in a private area within the ward to ensure confidentiality. Completed questionnaires were reviewed daily by the principal investigator for completeness and consistency to maintain data quality.

The questionnaire collected data on socio-demographic characteristics (age, sex, marital status, education level, employment and type of caregiver), clinical factors (psychiatric diagnosis, number of previous admissions in the past 12 months, comorbid medical conditions such as human immunodeficiency virus [HIV] or acquired immunodeficiency syndrome [AIDS], tuberculosis, diabetes mellitus, and hypertension, type of medication given at discharge), and behavioural factors (alcohol and substance use, treatment follow-up, drug adherent status and discharge against medical advice). Psychiatric diagnoses were obtained from patients’ medical records. For analysis, the psychosis category referred to non-schizophrenic psychotic disorders, including acute psychosis, puerperal psychosis, organic psychosis and substance-induced psychosis. Data on social support and history of physical and sexual abuse were also collected.

### Variables

The primary outcome variable was psychiatric readmission within 3 months of discharge. Participants were asked about previous admissions and provided the admission and discharge dates of their most recent hospitalisation. These dates were verified against hospital records. Patients with a new admission within 3 months of the recorded discharge date were classified as having psychiatric readmission.

Social support was assessed using a Multidimensional Scale of Perceived Social Support (MSPSS) adopted from Zimet et al.,^27^ a questionnaire consisting of 12 statements that explored emotional and practical support from family, friends and significant others. Participants were asked to rate their level of agreement with each statement on a five-point Likert scale, ranging from 1 (strongly disagree) to 5 (strongly agree). Overall score ranged from 12 to 60, and higher scores reflected stronger perceived social support from family, friends and significant others.

History of physical and sexual abuse was assessed using the psychosexual harassment questionnaire adapted from Sharifi Fard et al.^28^ The physical abuse section included six yes or no questions addressing experiences such as being hit, slapped, kicked, struck with objects or subjected to other forms of physical punishment causing injury or pain. The sexual abuse section included six yes or no questions exploring experiences of unwanted sexual contact, harassment, assault or coercion. Participants responded ‘Yes’ or ‘No’ to each item, with positive responses indicating a history of that specific form of abuse.

Drug adherence status was assessed using the Medication Adherence Rating Scale (MARS) adopted from Thompson et al.^29^ The instrument consists of 10 items with yes or no responses that measure both medication-taking behaviour and attitudes towards medication. Each ‘adherent’ response was scored as 1 and each ‘non-adherent’ response as 0, giving a possible total score ranging from 0 to 10. Adherence was evaluated based on the overall prescribed psychiatric treatment regimen, regardless of whether participants were taking a single medication or multiple medications. In this study, participants who scored ≥ 6 were categorised as adherent, while those scoring < 6 were categorised as non-adherent.

### Data analysis

The collected data were cleaned, coded and analysed using SPSS version 27.0 (IBM Corp., Armonk, New York, United States). Double data entry and consistency checks were performed to minimise entry errors. Descriptive statistics were used to summarise participant characteristics, with means and standard deviations for continuous variables, and frequencies and percentages for categorical variables. The prevalence of 3-month psychiatric readmission was calculated by dividing the number of patients readmitted within 3 months of their previous discharge by the total number of study participants.

Bivariate and multivariable analyses were conducted using modified Poisson regression with robust error variance to identify factors associated with psychiatric readmission. A total of 15 independent variables were examined in the bivariable analysis. Variables with a *p*-value < 0.20 (*n* = 11) were entered into the multivariable model to control for potential confounders. Treatment adherence status, number of previous admissions, comorbid medical conditions and alcohol or substance use had *p*-values > 0.20 and were therefore excluded from the multivariable analysis. Variables with a *p*-value < 0.05 in multivariable analysis were considered statistically significant.

### Ethical considerations

Ethical approval for the study was obtained from the Muhimbili University Institutional Review Board (IRB) (Ref. DA.282/298/01.C/2283), the Office of the Second Vice President of Zanzibar (Ref. CA.33/411/01M/14), and the ZAHRI (Ref. ZAHREC/02/ST/JUNE/2024/100). Participants were informed about the study purpose and procedures in a language and manner appropriate to their understanding, and written informed consent was obtained from their primary caregivers because of potential fluctuations in decision-making capacity. Data were anonymised and stored on a secure, password-protected computer, and physical documents were kept in a locked cabinet accessible only to the researchers.

## Results

### Characteristics of the study population

A total of 311 participants (mean age 36 years, s.d. ± 11 years), 52.4% female (*n* = 163), were enrolled. The majority of the participants were aged 18–29 years, 36.0% (*n* = 112), and nearly half of participants, 48.6% (*n* = 151) reported parents as their primary caregiver. Schizophrenia was the most common diagnosis, affecting 32.5% (*n* = 101) of participants. Detailed information on the sample characteristics is presented in Table 1.

**TABLE 1:** Characteristics of study population (*N* = 311).

Variable	Number	%
**Age (years)**		
18–29	112	36.0
30–39	91	29.3
40–49	74	23.8
50+	34	10.9
**Gender**		
Male	148	47.6
Female	163	52.4
**Marital status**		
Single	154	49.5
Married	87	28.0
Divorced or Widowed	70	22.5
**Patient occupation**		
Unemployed	203	65.3
Employed	108	34.7
**Education level**		
None	15	4.8
Primary	76	24.4
Secondary	175	56.3
Higher education	45	14.5
**Type of caregiver**		
Parents	151	48.6
Children	31	10.0
Siblings	78	25.1
Others	51	16.4
**Psychiatric diagnosis**		
Psychosis	88	28.3
Bipolar	51	16.4
Depression	27	8.7
Schizophrenia	101	32.5
Others (OCD, epilepsy)	44	14.1
**Social support**		
Inadequate	146	46.9
Adequate	165	53.1
**History of physical abuse**		
Yes	78	25.1
No	233	74.9
**History of sexual abuse**		
Yes	44	14.1
No	267	85.9
**Type of medication given during last discharge**		
Oral medication	221	71.3
Both (oral and injection)	89	28.7
**Treatment adherence status**		
Adherent	235	75.6
Non-adherent	76	24.4
**Number of previous admissions**		
2 or less	202	65.0
3 or more	109	35.0
**Comorbid medical condition**		
None	301	96.8
One or more comorbidities	10	3.2
**Alcohol or substance use**		
Yes	48	15.4
No	263	84.6

OCD, obsessive complusive disorders.

### Prevalence of psychiatric readmission

Of the 311 participants, 182 were readmitted within 3 months. The prevalence of psychiatric readmission was 58.5% (95% confidence interval [CI]: 52.9% – 63.9%). Readmission was more common among males at 64.9% compared to females at 52.8% (*p*-value = 0.030) (Figure 1), and highest among those aged 30–39 years at 71.4%, while the lowest prevalence was observed in the 18–29 years group at 45.5% (*p*-value = 0.002).

**FIGURE 1:**
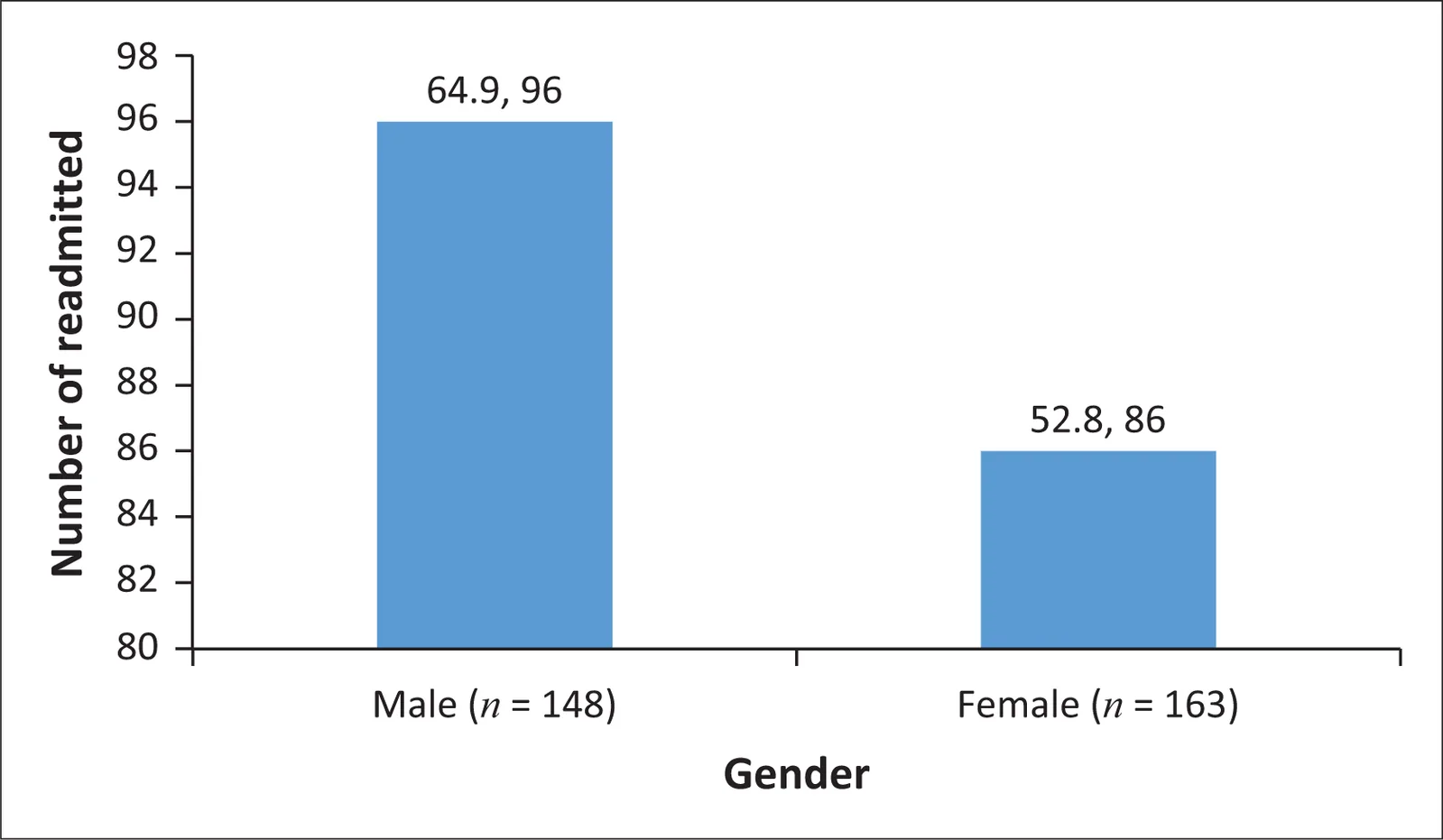
Prevalence of psychiatric readmission within 3 months by gender (%, *n*) (*N* = 311).

### Factors associated with readmission within 3 months

The modified multivariable Poisson regression results revealed that individuals aged 30–39 years had the highest risk of readmission (adjusted prevalence ratio [APR] = 1.47, 95% CI: 1.16–1.84, *p*-value = 0.001) compared to those aged 20–29 years. Patients with a diagnosis of schizophrenia (APR = 1.23, 95% CI: 1.00–1.50, *p*-value = 0.041) and bipolar disorders (APR = 1.69, 95% CI: 1.26–2.26, *p*-value < 0.001) showed an increased risk of readmission compared to psychosis. Patients with higher education were also more likely to be readmitted compared to those with no formal education (APR = 1.61, 95% CI: 1.16–2.22, *p*-value = 0.010). Additionally, physically abused individuals had a significantly higher risk of readmission (APR = 1.49, 95% CI: 1.16–2.22, *p*-value < 0.001) compared to those without such a history. Patients who received both oral and injectable medications also had an increased risk of readmission (APR = 1.46, 95% CI: 1.37–2.20, *p*-value < 0.001) (Table 2). Patients cared for by their children were less likely to be readmitted (APR = 0.38, 95% CI: 0.16–0.89, *p*-value = 0.025) compared to those cared for by parents. Detailed findings are summarised in Table 2.

**TABLE 2:** Bivariable and multivariable modified Poisson regression analysis results for factors associated with psychiatric readmission.

Predictor	Total	Number readmitted	%	Bivariable			Multivariable		
				CPR	95% CI	*p*-value	APR	95% CI	*p*-value
**Age categories (years)**									
18–29	112	51	45.5	Ref.	Ref.	-	Ref.	Ref.	-
30–39	91	65	71.4	1.56	1.23–1.99	< 0.001	1.47	1.16–1.84	0.001
40–49	74	43	58.1	1.27	0.96–1.68	0.088	1.24	0.96–1.59	0.100
50+	34	23	67.6	1.48	1.09–2.02	0.012	1.42	0.92–2.02	0.066
**Gender**									
Female	163	86	52.8	Ref.	Ref.	-	Ref.	Ref.	-
Male	148	96	64.9	1.22	1.01–1.48	0.031	0.88	0.74–1.06	0.196
**Marital status**									
Single	154	100	64.9	Ref.	Ref.	-	Ref.	Ref.	-
Married	87	52	59.8	0.92	0.74–1.13	0.434	0.92	0.73–1.16	0.496
Divorced or Widowed	70	30	42.9	0.66	0.49–0.88	0.006	0.82	0.59–1.13	0.242
**Education level**									
None	15	6	40.0	Ref.	Ref.	-	Ref.	Ref.	-
Primary	76	35	46.1	1.15	0.59–2.24	0.678	1.60	0.99–2.59	0.052
Secondary	175	108	61.7	1.54	0.82–2.89	0.178	1.62	1.04–2.51	0.032
Higher education	45	33	73.3	1.83	0.96–3.49	0.065	1.88	1.16–3.03	0.010
**Caregiver**									
Parents	151	86	57.0	Ref.	Ref.	-	Ref.	Ref.	-
Children	31	6	19.4	0.34	0.16–0.70	0.004	0.38	0.16–0.89	0.025
Siblings	78	62	79.5	1.39	1.16–1.66	< 0.001	1.08	0.87–1.35	0.438
Others	51	28	54.9	0.96	0.72–1.28	0.801	0.89	0.71–1.12	0.248
**Occupation**									
Unemployed	203	110	54.2	Ref.	Ref.	-	Ref.	Ref.	-
Employed	108	72	66.2	1.23	1.02–1.47	0.027	1.29	1.06–1.65	0.009
**Psychiatric diagnosis**									
Psychosis	88	45	51.1	Ref.	Ref.	-	Ref.	Ref.	-
Bipolar	51	37	72.5	1.41	1.08–1.84	0.010	1.69	1.26–2.26	< 0.001
Depression	27	5	18.5	0.36	0.16–0.82	0.015	0.52	0.22–1.23	0.139
Schizophrenia	101	70	69.3	1.35	1.06–1.72	0.014	1.23	1.00–1.50	0.041
Others (OCD, epilepsy)	44	25	56.8	1.11	0.80–1.84	0.530	1.13	0.79–1.60	0.491
**History of physical abuse**									
No	233	115	48.4	Ref.	Ref.	-	Ref.	Ref.	-
Yes	78	67	85.9	1.74	1.48–2.03	< 0.001	1.49	1.22–1.83	< 0.001
**History of sexual abuse**									
No	267	150	56.2	Ref.	Ref.	-	Ref.	Ref.	-
Yes	44	32	72.7	1.29	1.05–1.59	0.016	1.16	0.90–1.50	0.227
**Type of medication**									
Oral	221	101	45.7	Ref.	Ref.	-	Ref.	Ref.	-
Oral and Injection	89	81	91.0	1.99	1.70–2.33	< 0.001	1.46	1.46–2.20	< 0.001
**Social support**									
Adequate	165	109	66.1	Ref.	Ref.	-	Ref.	Ref.	-
Inadequate	146	73	50	0.75	0.62–0.92	0.005	0.80	0.80–1.13	0.605
**Treatment adherence status**									
Adherent	235	133	56.6	Ref.	Ref.	-	-	-	-
Non-adherent	76	49	64.5	1.13	0.93–1.39	0.204	-	-	-
**Number of previous admissions**									
2 or less	202	114	56.4	Ref.	Ref.	-	-	-	-
3 or more	109	68	62.4	1.10	0.91–1.33	0.300	-	-	-
**Comorbid medical condition**									
None	301	176	58.4	Ref.	Ref.	-	-	-	-
One or more comorbidities	10	6	60.0	1.02	0.61–1.71	0.922	-	-	-
**Substance use**									
No	263	151	57.4	Ref.	Ref.	-	-	-	-
Yes	48	31	64.5	1.12	0.89–1.42	0.324	-	-	-

CPR, crude prevalence ratio; APR, adjusted prevalence ratio; CI, confidence interval; OCD, obsessive complusive disorders; Ref., reference.

## Discussion

In this study of readmission among psychiatric patients admitted at a tertiary psychiatric hospital in Zanzibar, a high prevalence of 3-month psychiatric readmission was observed. Factors associated with increased risk of psychiatric readmission were middle-aged adults, being employed and having secondary or higher education, having schizophrenia and bipolar as diagnosis type, history of physical abuse and being prescribed a combination of oral and injectable medications.

The readmission rate in Zanzibar is substantially higher compared to other countries. In the United States, readmission rates within 3 months range from 12%^30^ to 21%,^31^ in South Africa, Behr et al.^32^ reported a readmission rate of 21%. This finding resembles Miller et al.^33^ in Canada, who also reported a similarly high rate of 57.3%, suggesting vulnerability to relapse even in settings with more advanced resources. Variations in readmission rates across countries can largely be attributed to differences in healthcare systems, including access to mental health services, availability of medications, community-based care programmes and follow-up mechanisms.

In Tanzania, persistent challenges such as underinvestment, shortage of mental healthcare providers, inadequate infrastructure and the absence of a standalone mental health policy may explain the higher readmission rates observed in this study.^34,35^ These challenges underscore the urgent need for increased investment in mental health services, which currently receive only 4% of the national health budget,^35^ and developing a dedicated policy framework to provide strategic direction and strengthen accountability.^36^ Evidence from neighbouring countries, such as Kenya, shows that investing in mental health yields long-term health, social and economic benefits,^37^ suggesting similar potential gains for Tanzania.

The study showed that patients aged 30–39 years had a higher risk of readmission compared to the youngest group (18–29 years). This finding resembles Everet et al.^19^ in 2022 in Pennsylvania, who also reported high readmission rates among these patients. Higher relapses and hospitalisation rates in this age group may be because of cumulative illness burden and life stressors.^38,39^ Patients cared for by children had a much lower risk of readmission compared to those cared for by parents. This finding suggests that adult children may provide more consistent and supportive monitoring than parents.^40^ However, this finding may reflect underlying differences in patient characteristics, as parents may be more likely to care for middle-aged individuals who already have a higher risk of readmission. This study urges the need to actively involve adult children in the patient’s care plan, engaging them as integral caregivers can strengthen adherence, enhance early detection of relapse and ultimately reduce the risk of psychiatric readmission.

Patients with higher education were more likely to be readmitted than those with no formal education. This finding differs from previous studies reporting that higher educational attainment is generally associated with better health outcomes.^41,42^ One possible explanation is that individuals with higher education may be more likely to recognise early signs of relapse and seek readmission promptly when symptoms recur.^42^ In addition, they may return to work or academic activities sooner after discharge, where increased responsibilities and stress could contribute to symptom exacerbation and the need for readmission.^43,44^ Further studies are needed to understand the mechanisms underlying this relationship.

Patients receiving both oral and injectable medications had a significantly higher risk of readmission than those receiving oral medication alone. This association likely reflects greater illness severity rather than the effect of the medication given, as combination therapy is often prescribed for patients with recurrent relapses, poor response to treatment or difficulties with medication adherence.^45,46^ Complex treatment regimens and antipsychotic-related adverse effects may further contribute to inconsistent adherence, increasing the risk of symptom exacerbation and subsequent hospitalisation.^24,47^ These findings highlight the importance of individualised discharge planning, adherence support and close post-discharge follow-up for patients requiring combined treatment.

In this study, one quarter of participants reported a history of physical abuse, and these patients had nearly twice the risk of psychiatric readmission compared to those without such a history. This finding aligns with previous research showing that trauma and abuse have long-term psychological effects that can worsen psychiatric symptoms, reduce coping ability and increase vulnerability to relapse and hospitalisation.^30,31^ Similarly, patients diagnosed with bipolar disorder and schizophrenia were at the highest risk of readmission. Similar findings were reported by Stewart et al.^14^ and Caseiro et al.,^45^ who also reported higher readmission rates among patients. The recurrent nature of bipolar disorder and the chronic course of schizophrenia, often accompanied by challenges in treatment adherence, contribute to frequent relapses and hospitalisations.^45,46,48^ These findings underscore the need for trauma-informed care, closer monitoring and tailored follow-up strategies for patients with severe mental disorders to improve long-term outcomes and reduce repeated admissions.

In this study, 15.4% of participants reported a history of drug use. This proportion was lower than that reported in previous studies from Tanzania (32.7%) and Botswana (85.1%).^49,50^ The difference may partly be explained by variations in the definition and assessment methods. Both of these studies used the Alcohol, Smoking and Substance Involvement Screening Test (ASSIST), which provides a comprehensive assessment of lifetime and recent substance use as well as substance-related problems,^51^ and may therefore identify more individuals with previous substance exposure, whereas the present study assessed only drug use within the last 3 months.

The present study did not find a significant association between substance use and psychiatric readmission. This finding contrasts with previous studies that identified substance use as a risk factor for psychiatric readmission.^8,19^ The absence of an association in the present study may be explained by the relatively small number of participants reporting a history of drug use, which may have limited the statistical power to detect a significant effect.^52,53^ However, given the well-established association between substance use and adverse psychiatric outcomes,^8,19^ routine screening and appropriate management of substance use should remain integral components of psychiatric care.

This study has some limitations that should be considered. Data were collected from a single tertiary psychiatric facility, which may limit generalisability to other settings. In addition, the use of convenience sampling could introduce selection bias, as a result, certain groups of patients may have been overrepresented, while others may have been underrepresented. This limits the ability to generalise the findings to the broader population of psychiatric patients. Future research should include multiple facilities and employ more representative sampling methods. Moreover, qualitative studies exploring patient experiences, healthcare provider perspectives and systemic challenges in psychiatric care could provide deeper insights into factors influencing readmission.

## Conclusion

The prevalence of psychiatric readmission within 3 months was found to be high, underscoring a significant burden on both the healthcare system and affected patients. These findings highlight the urgent need to strengthen mental healthcare services and post-admission care. Interventions should focus on high-risk groups, including middle-aged individuals, patients diagnosed with schizophrenia and bipolar disorders, patients with a history of physical abuse, higher education and those requiring combination treatment to break the cycle of readmission and promote long-term mental health recovery.

## References

[1] World Health Organization (WHO). Special initiative for mental health (2019–2023) [homepage on the Internet]. 2019 [cited 2023 Sep 28]. Available from: https://www.who.int/publications/i/item/special-initiative-for-mental-health-(2019-2023)

[2] World Health Organization (WHO). Mental health Atlas 2020 country profile: France [homepage on the Internet]. 2020 [cited 2025 Oct 13]. Available from: https://datahelpdesk.worldbank.org/knowledgebase/topics/19280-country-classification

[3] World Health Organization (WHO). Mental health Atlas 2020 country profile: Kenya [homepage on the Internet]. 2020 [cited 2025 Oct 13]. Available from: https://datahelpdesk.worldbank.org/knowledgebase/topics/19280-country-classification

[4] World Health Organization (WHO). Mental health Atlas 2017 [homepage on the Internet]. WHO; 2017 [cited 2024 Feb 05]. Available from: https://www.who.int/publications/m/item/mental-health-atlas-2017-country-profile-united-republic-of-tanzania

[5] Moitra M, Santomauro D, Collins PY, et al. The global gap in treatment coverage for major depressive disorder in 84 countries from 2000–2019: A systematic review and Bayesian meta-regression analysis. PLoS Med. 2022;19(2):e1003901. 10.1371/journal.pmed.1003901 PMC8846511 35167593

[6] Lai YM, Hong CP, Chee CY. Stigma of mental illness. Singapore Med J [serial online]. 2001 [cited 2023 Dec 21];42(3):111–114. Available from: http://www.ncbi.nlm.nih.gov/pubmed/11405561 11405561

[7] Lassemo E, Myklebust LH, Salazzari D, Kalseth J. Psychiatric readmission rates in a multi-level mental health care system – A descriptive population cohort study. BMC Health Serv Res. 2021;21(1):378. 10.1186/s12913-021-06391-7 PMC8067649 33892715

[8] Owusu E, Oluwasina F, Nkire N, Lawal MA, Agyapong VIO. Readmission of patients to acute psychiatric hospitals: Influential factors and interventions to reduce psychiatric readmission rates. Healthcare (Switzerland). 2022;10(9):1808. 10.3390/healthcare10091808 PMC9498532 36141418

[9] Pourat N, Chen X, Wu SH, Davis AC. Timely outpatient follow-up is associated with fewer hospital readmissions among patients with behavioral health conditions. J Am Board Fam Med. 2019;32(3):353–361. 10.3122/jabfm.2019.03.180244 31068399

[10] Zhou Y, Ning Y, Fan N, Mohamed S, Rosenheck RA, He H. Correlates of readmission risk and readmission days in a large psychiatric hospital in Guangzhou, China. Asia Pac Psychiatry. 2014;6(3):342–349. 10.1111/appy.12096 24038857

[11] Behr GM, Christie C, Soderlund N, Lee T. Patterns and determinants of acute psychiatric readmissions. S Afr Med J. 2002;92(5):369–374. 12108169

[12] Abaatyo J, Kaggwa MM, Favina A, Olagunju AT. Readmission and associated clinical factors among individuals admitted with bipolar affective disorder at a psychiatry facility in Uganda. BMC Psychiatry. 2023;23(1):474. 10.1186/s12888-023-04960-0 PMC10308791 37380963

[13] Bobier C, Bobier C, Warwick M. Factors associated with readmission to adolescent psychiatric care. Aust N Z J Psychiatry. 2005;39(7):600–606. 10.1080/j.1440-1614.2005.01632.x 15996141

[14] Stewart SL, Kam C, Baiden P. Predicting length of stay and readmission for psychiatric inpatient youth admitted to adult mental health beds in Ontario, Canada. Child Adolesc Ment Health. 2014;19(2):115–121. 10.1111/camh.12022 32878390

[15] Green JH. Frequent rehospitalization and noncompliance with treatment. Psychiatr Serv. 1988;39(9):963–966. 10.1176/ps.39.9.963 3215645

[16] Fikreyesus M, Soboka M, Feyissa GT. Psychotic relapse and associated factors among patients attending health services in Southwest Ethiopia: A cross-sectional study. BMC Psychiatry. 2016;16(1):354. 10.1186/s12888-016-1076-2 PMC5072324 27765033

[17] Sariah AE, Outwater AH, Malima KIY. Risk and protective factors for relapse among Individuals with Schizophrenia: A qualitative study in Dar es Salaam, Tanzania. BMC Psychiatry. 2014;14(1):240. 10.1186/s12888-014-0240-9 PMC4169829 25168715

[18] Mlada K, Formanek T, Vevera J, Latalova K, Winkler P, Volavka J. Serious physical assault and subsequent risk for rehospitalization in individuals with severe mental illness: A nationwide, register-based retrospective cohort study. Ann Gen Psychiatry. 2021;20(1):44. 10.1186/s12991-021-00358-y PMC8449895 34537054

[19] Everett J, Druyor K, Krasinski C, Obaid M, Li Y. Predictors of behavioral health unit readmission within 30 days of discharge: A retrospective study. Heliyon. 2022;8(10):e10784. 10.1016/j.heliyon.2022.e10784 PMC9547231 36217492

[20] Samuel T, Nigussie K, Mirkena Y, Azale T. Relationship between social support and schizophrenia relapse among patients with schizophrenia on follow-up at Amanuel Mental Specialized Hospital, Addis Ababa, Ethiopia: A case-control study. Front Psychiatry. 2022;13:980614. 10.3389/fpsyt.2022.980614 PMC9730020 36506425

[21] Gbiri CA, Badru FA, Ladapo HTO, Gbiri AA. Socio-economic correlates of relapsed patients admitted in a Nigerian mental health institution. Int J Psychiatry Clin Pract. 2011;15(1):19–26. 10.3109/13651501.2010.506954 22122685

[22] Choi M, Kim H, Qian H, Palepu A. Readmission rates of patients discharged against medical advice: A matched cohort study. PLoS One. 2011;6(9):e24459. 10.1371/journal.pone.0024459 PMC3169593 21931723

[23] Haji Kassim KK, Haji Md Safar MSB, Lupat A, Zolkefli Y. Mental health nurses’ views of ward readmission: A focus group study in Brunei Darussalam. Belitung Nurs J. 2021;7(5):402–408. 10.33546/bnj.1666 PMC10367975 37496506

[24] Iseselo MK, Ambikile JS. Medication challenges for patients with severe mental illness: Experience and views of patients, caregivers and mental health care workers in Dar es Salaam, Tanzania. Int J Ment Health Syst. 2017;11(1):17. 10.1186/s13033-017-0126-6 PMC5294727 28184242

[25] Sveticic J, Turner K, Bethi S, et al. Short stay unit for patients in acute mental health crisis: A case-control study of readmission rates. Asia Pac Psychiatry. 2020;12(1):e12376. 10.1111/appy.12376 31883230

[26] Thinn DSS, Kuswanto CN, Sum MY, et al. The 23-hour observation unit admissions within the emergency service at a National Tertiary Psychiatric Hospital. Prim Care Companion CNS Disord. 2015;17(4):15m01789. 10.4088/PCC.15m01789 PMC4664576 26693048

[27] Zimet GD, Dahlem NW, Zimet SG, Farley GK. The multidimensional scale of perceived social support. J Pers Assess. 1988;52(1):30–41. 10.1207/s15327752jpa5201_2 2280326

[28] Sharifi Fard SA, Hasanvand F, Ahmadpanah M, Zoghi Paidar MR, Kazemi Z, Parchami Khorram M. Design and validation of the psychosexual harassment q‘uestionnaire. J Inj Violence Res [serial online]. 2023 [cited 2025 Oct 02];15(1):63–82. Available from: https://pmc.ncbi.nlm.nih.gov/articles/PMC10369331/10.5249/jivr.v15i1.1777 PMC10369331 36609357

[29] Thompson K, Kulkarni J, Sergejew AA. Reliability and validity of a new Medication Adherence Rating Scale (MARS) for the psychoses. Schizophr Res. 2000;42(3);241–247. 10.1016/S0920-9964(99)00130-9 10785582

[30] Joyce VW, King CD, Nash CC, Lebois LAM, Ressler KJ, Buonopane RJ. Predicting psychiatric rehospitalization in adolescents. Adm Policy Ment Health. 2019;46(6):807–820. 10.1007/s10488-019-00982-7 PMC6886255 31584109

[31] Czyz EK, Berona J, King CA. Rehospitalization of suicidal adolescents in relation to course of suicidal ideation and future suicide attempts. Psychiatr Serv. 2016;67(3):332–338. 10.1176/appi.ps.201400252 PMC5037573 26725287

[32] Behr GM, Christie C, Soderlund N, Lee T. Patterns and determinants of acute psychiatric readmissions. S Afr J Psychiatry. 2002;8(3):a930. 10.4102/sajpsychiatry.v8i3.930 12108169

[33] Miller DAA, Ronis ST, Slaunwhite AK, et al. Longitudinal examination of youth readmission to mental health inpatient units. Child Adolesc Ment Health. 2020;25(4):238–248. 10.1111/camh.12371 32516481

[34] Van Rensburg AJ, Fourie P. Health policy and integrated mental health care in the SADC region: Strategic clarification using the Rainbow Model. Int J Ment Health Syst. 2016;10(1):49. 10.1186/s13033-016-0081-7 PMC4957874 27453722

[35] World Health Organization (WHO). Mental health Atlas 2020 country profile: United Republic of Tanzania [homepage on the Internet]. 2020 [cited 2025 Sep 28]. Available from: https://www.who.int/publications/m/item/mental-health-atlas-tza-2020-country-profile

[36] World Health Organization (WHO). Guidance on mental health policy and strategic action plans: Module 1. Introduction, purpose and use of the guidance. Geneva: WHO; 2025.

[37] RoK Ministry of Health. Kenya mental health investment case 2021: Providing evidence for the long-term health, social, and economic benefits of investment in mental health in Kenya [homepage on the Internet]. 2021 [cited 2025 Sep 28]. Available from: http://www.health.go.ke

[38] Soreca I, Fagiolini A, Frank E, Houck PR, Thompson WK, Kupfer DJ. Relationship of general medical burden, duration of illness and age in patients with bipolar I disorder. J Psychiatr Res. 2008;42(11):956–961. 10.1016/j.jpsychires.2007.10.009 18076906

[39] Librero J, Peiró S, Ordiñana R. Chronic comorbidity and outcomes of hospital care. J Clin Epidemiol. 1999;52(3):171–179. 10.1016/S0895-4356(98)00160-7 10210233

[40] Ghosh S, Greenberg J. Aging fathers of adult children with schizophrenia: The toll of caregiving on their mental and physical health. Psychiatr Serv. 2009;60(7):982–984. 10.1176/ps.2009.60.7.982 19564232

[41] Cutler D, Lleras-Muney A. Education and health: Evaluating theories and evidence. Cambridge, MA: National Bureau of Economic Research; 2006.

[42] Berkman ND, Sheridan SL, Donahue KE, Halpern DJ, Crotty K. Low health literacy and health outcomes: An updated systematic review. Ann Intern Med. 2011;155(2):97–107. 10.7326/0003-4819-155-2-201107190-00005 21768583

[43] Nielsen MBD, Madsen IEH, Bultmann U, Christensen U, Diderichsen F, Rugulies R. Predictors of return to work in employees sick-listed with mental health problems: Findings from a longitudinal study. Eur J Public Health. 2011;21(6):806–811. 10.1093/eurpub/ckq171 21126986

[44] Nigatu YT, Liu Y, Uppal M, et al. Prognostic factors for return to work of employees with common mental disorders: A meta-analysis of cohort studies. Soc Psychiatry Psychiatr Epidemiol. 2017;52(10):1205–1215. 10.1007/s00127-017-1402-0 28577211

[45] Caseiro O, Pérez-Iglesias R, Mata I, et al. Predicting relapse after a first episode of non-affective psychosis: A three-year follow-up study. J Psychiatr Res. 2012;46(8):1099–1105. 10.1016/j.jpsychires.2012.05.001 22721546

[46] Lauber C, Keller C, Eichenberger A, Rössler W. Family burden during exacerbation of schizophrenia: Quantification and determinants of additional costs. Int J Soc Psychiatry. 2005;51(3):259–264. 10.1177/0020764005057376 16252793

[47] Muench J, Hamer AM. Adverse effects of antipsychotic medications. Am Fam Physician [serial online]. 2010 [cited 2025 Jan 08];81(5):617–622. Available from: www.aafp.org/afpAmericanFamilyPhysician617 20187598

[48] Clemente AS, Diniz BS, Nicolato R, et al. Bipolar disorder prevalence: A systematic review and meta-analysis of the literature. Rev Bras Psiquiatr. 2015;37(2):155–161. 10.1590/1516-4446-2012-1693 25946396

[49] Otlhapile OE, Gitau CW, Kuria MW. The prevalence of substance use disorders and associated characteristics among patients admitted to a psychiatric hospital in Botswana. Int J Psychiatry Med. 2023;58(4):339–357. 10.1177/00912174231156027 36752226

[50] Karino K, Ambikile JS, Iseselo MK. Prevalence and factors associated with self-reported substance use among patients with mental illness in Dar es Salaam, tanzania: A cross-sectional analytical study. Mental Illness. 2023;2023:1–8. 10.1155/2023/3923793

[51] Humeniuk R. The Alcohol, smoking and substance involvement screening test (ASSIST): Manual for use in primary care. Geneva: World Health Organization; 2010.

[52] Vittinghoff E, McCulloch CE. Relaxing the rule of ten events per variable in logistic and Cox regression. Am J Epidemiol. 2007;165(6):710–718. 10.1093/aje/kwk052 17182981

[53] Peduzzi P, Concato J, Kemper E, Holford TR, Feinstein AR. A simulation study of the number of events per variable in logistic regression analysis. J Clin Epidemiol. 1996;49(12):1373–1379. 10.1016/S0895-4356(96)00236-3 8970487

